# Identification of m5C-Related gene diagnostic biomarkers for sepsis: a machine learning study

**DOI:** 10.3389/fgene.2024.1444003

**Published:** 2024-10-30

**Authors:** Siming Lin, Kexin Cai, Shaodan Feng, Zhihong Lin

**Affiliations:** ^1^ Department of Emergency Medicine, The First Affiliated Hospital of Fujian Medical University, Fuzhou, China; ^2^ Department of Emergency Medicine, National Regional Medical Center, Binhai Campus of the First Affiliated Hospital, Fujian Medical University, Fuzhou, China

**Keywords:** bioinformatics, diagnostic biomarkers, immune infiltration, machine learning, m5C-related gene, sepsis

## Abstract

**Background:**

Sepsis is a serious condition that occurs when the body’s response to infection becomes uncontrolled, resulting in a high risk of death. Despite improvements in healthcare, identifying sepsis early is difficult because of its diverse nature and the absence of distinct biomarkers. Recent studies suggest that 5-methylcytosine (m5C)-related genes play a significant role in immune responses, yet their diagnostic potential in sepsis remains unexplored.

**Methods:**

This research combined and examined four sepsis-related datasets (GSE95233, GSE57065, GSE100159, and GSE65682) sourced from the Gene Expression Omnibus (GEO)database to discover m5C-related genes with differential expression. Various machine learning methods, such as decision tree, random forest, and XGBoost, were utilized in identifying crucial hub genes. Receiver Operating Characteristic (ROC) curve analysis was used to assess the diagnostic accuracy of these genetic markers. Additionally, single-gene enrichment and immune infiltration analyses were conducted to investigate the underlying mechanisms involving these hub genes in sepsis.

**Results:**

Three hub genes, DNA Methyltransferase 1 (*DNMT1*), tumor protein P53 (*TP53*), and toll-like receptor 8 (*TLR8*), were identified and validated for their diagnostic efficacy, showing area under the curve (AUC) values above 0.7 in both test and validation sets. Enrichment analyses revealed that these genes are involved in key pathways such as p53 signaling and Toll-like receptor signaling. Immune infiltration analysis indicated significant correlations between hub genes and various immune cell types, suggesting their roles in modulating immune responses during sepsis.

**Conclusion:**

The study highlights the diagnostic potential of m5C-related genes in sepsis and their involvement in immune regulation. These findings offer new insights into sepsis pathogenesis and suggest that *DNMT1*, *TP53*, and *TLR8* could serve as valuable biomarkers for early diagnosis. Further studies should prioritize validating these biomarkers in clinical settings and investigating their potential for therapy.

## 1 Introduction

Sepsis is a serious condition caused by an uncontrolled reaction of the body to an infection, resulting in organ failure and a high risk of death (Singer et al., 2016). The Global Burden of Disease Study reports that sepsis impacts around 49 million people each year and results in 11 million deaths globally, making up almost a fifth of all worldwide fatalities (Rudd et al., 2020). Even with improvements in healthcare, identifying and treating sepsis continues to be difficult because of its diverse characteristics and the absence of distinct biomarkers (Seymour et al., 2016; Singer et al., 2016). Current diagnostic methods rely heavily on clinical criteria and laboratory tests that often lack sensitivity and specificity, leading to delays in diagnosis and treatment (Gotts and Matthay, 2016). Hence, it is imperative to develop new indicators that can enhance the timely detection and prediction of sepsis.

Recent research has emphasized the significance of epigenetic changes, like methylation, in the development of different illnesses, including sepsis (Bomsztyk et al., 2015; van der Poll et al., 2017). One of the changes that has received focus is m5C, which is known for its control over gene expression and immune system reactions (Wnuk et al., 2020; Qin et al., 2021; Zhang et al., 2022; Tian et al., 2023). Studies have shown that genes related to m5C play a role in controlling the function of immune cells and the body’s inflammatory reactions, both of which are crucial in the advancement of sepsis (Medzhitov, 2008; Angus and van der Poll, 2013). In cancer studies, m5C has been demonstrated to impact the tumor microenvironment and infiltration of immune cells (Zhang et al., 2020; Song et al., 2022; Yu et al., 2022; Gu et al., 2023; Zhang et al., 2023), indicating its possible involvement in regulating immune reactions during sepsis. However, the diagnostic potential and immunological implications of m5C-related genes in sepsis remain largely unexplored.

This study aimed to explore the diagnostic effectiveness and immune infiltration features of m5C-associated genes in cases of sepsis. To identify m5C-related genes and their associated biological pathways, we examined four sepsis-related datasets (GSE95233, GSE57065, GSE100159, and GSE65682) obtained from the GEO database through integration and analysis. Machine learning techniques were employed to select key hub genes, and their diagnostic performance was evaluated using ROC curve analysis. Additionally, we conducted an analysis of individual genes to identify enrichment and immune infiltration, aiming to understand the possible mechanisms that contribute to the role of these central genes in sepsis.

By providing a comprehensive analysis of m5C-related genes in sepsis, our study aims to uncover novel biomarkers that can enhance the early diagnosis and understanding of the immunological landscape of this complex disease.

## 2 Materials and methods

### 2.1 Acquisition and merging of datasets

The datasets GSE95233, GSE57065, GSE100159, and GSE65682 related to sepsis were obtained from the GEO database (https://www.ncbi.nlm.nih.gov/geo/) (Table 1). The data platforms of GSE95233 and GSE57065 were GPL570. GSE95233 contained 22 normal samples and 102 sepsis samples. In GSE57065, there are 25 samples classified as normal and 82 samples classified as sepsis. These two datasets were combined using the R package inSilicoMerging, followed by the application of the COMBAT method to eliminate batch effects. The merged dataset was used as the test set. The GSE100159 data platform was GPL6884, which contained 12 normal samples and 35 sepsis samples. The GSE65682 data platform was GPL13667, which contained 42 normal samples and 479 sepsis samples. The required samples were extracted as the validation set, respectively.

**TABLE 1:** Information on selected datasets.

Dataset	Normal (N)	Sepsis (N)	Platform	Attribute
GSE95233	22	102	GPL570	Test set
GSE57065	25	82		
GSE100159	12	35	GPL6884	Validation set
GSE65682	42	479	GPL13667	

### 2.2 Difference analysis and enrichment analysis

According to the information on sample grouping, the groups were analyzed differentially using the limma package in R. The genes with adjusted *p*-value <0.05 and |logFC| > 0.263 (equivalent to a 1.2-fold difference) were identified as differentially expressed genes (DEGs). The differentially expressed genes were visualized using volcano plots created with the ggplot2 package in R. A total of 48 m5C-related genes were gathered from GeneCards (https://www.genecards.org/). The overlap between the differentially expressed genes and m5C-related genes was determined using a Venn diagram, resulting in m5C-related differential genes. These m5C-related differential genes were then depicted in a heatmap using the R package pheatmap. The clusterProfiler package in R was utilized to conduct gene ontology (GO) functional annotation analysis and Kyoto Encyclopedia of Genes and Genomes (KEGG) pathway enrichment analysis for m5C-related differential genes.

### 2.3 Machine learning screening of hub genes

In order to screen the most diagnostically significant m5C-related genes, we used R’s rpart package for decision tree analysis, the randomForest package for random forest analysis, and the xgboost package for XGBOOST analyses. We then performed importance analysis on the included genes, respectively. We selected only the top 5 genes in importance from each method and used the common genes among them as hub genes.

### 2.4 Analysis and validation of hub genes diagnostic efficacy

ROC analysis was performed using the pROC package in R. The results were visualized using ggplot2 to assess the diagnostic efficacy of the hub gene, with an AUC >0.7 indicating high diagnostic efficacy. The diagnostic efficacy of the hub gene was verified using GSE100159 and GSE65682.

### 2.5 Single gene enrichment analysis of hub genes

We obtained the Gene Set Enrichment Analysis (GSEA) software (version 3.0) from the website of GSEA (http://software.broadinstitute.org/gsea/index.jsp) and divided the samples into a high expression group (≥50%) and a low expression group (<50%) according to the expression level of the hub gene. We downloaded the c2. cp.kegg.v7.4. symbols.gmt subset from the Molecular Signatures Database (http://www.gsea-msigdb.org/gsea/downloads.jsp) to assess relevant pathways and molecular mechanisms. During GSEA analysis, based on gene expression profiles and phenotypic grouping, we set a minimum gene set of 5 and a maximum gene set of 5,000. One thousand resamplings were performed, and a *p*-value <0.05 and a false discovery rate (FDR) < 0.25 were considered statistically significant.

### 2.6 Immune infiltration analysis

To further explore the similarities and differences in the levels of immune cell infiltration between the two groups of samples, we uploaded the merged dataset (GSE95233 and GSE57065) to CIBERSORTx (https://cibersortx.stanford.edu/) and analyzed the immune cell infiltration using LM22 as the reference dataset, with the cutoff point set to a *p*-value <0.05. We plotted bar graphs in R to illustrate the percentage of each immune cell type in individual samples, box line plots to show the infiltration of all immune cells under different grouping scenarios, and correlation plots to show correlation plots between each immune cell and each hub gene. In addition, to directly view the correlation between hub genes and immune cell infiltration levels, correlation scatter plots were generated, and correlation curves were fitted for gene-immune cell pairs with significant correlations (correlation coefficients greater than 0.6).

### 2.7 Statistical analyses

Data processing and analysis were conducted using Excel (Microsoft) and R software (version 4.2.1). To compare two sets of continuous variables, the independent Student’s t-test was used to determine statistical significance for normally distributed variables, while the Mann-Whitney U test (also known as the Wilcoxon rank sum test) was used for non-normally distributed variables. Either the chi-square test or Fisher’s exact test was utilized for comparing and analyzing the statistical significance of two groups of categorical variables. The Kruskal–Wallis test was utilized for comparing multiple groups, while the Wilcoxon test was employed for comparing two groups. A two-tailed *p*-value <0.05 was considered statistically significant.

### 2.8 Ethics statement

No ethical approval was necessary for the human studies as the data from the GEO database is easily accessible to the public. Participants or their legal guardians/next of kin were not required to provide written informed consent to take part in this study, as per national laws and institutional guidelines.

## 3 Results

### 3.1 Data pre-processing

**FIGURE 1:**
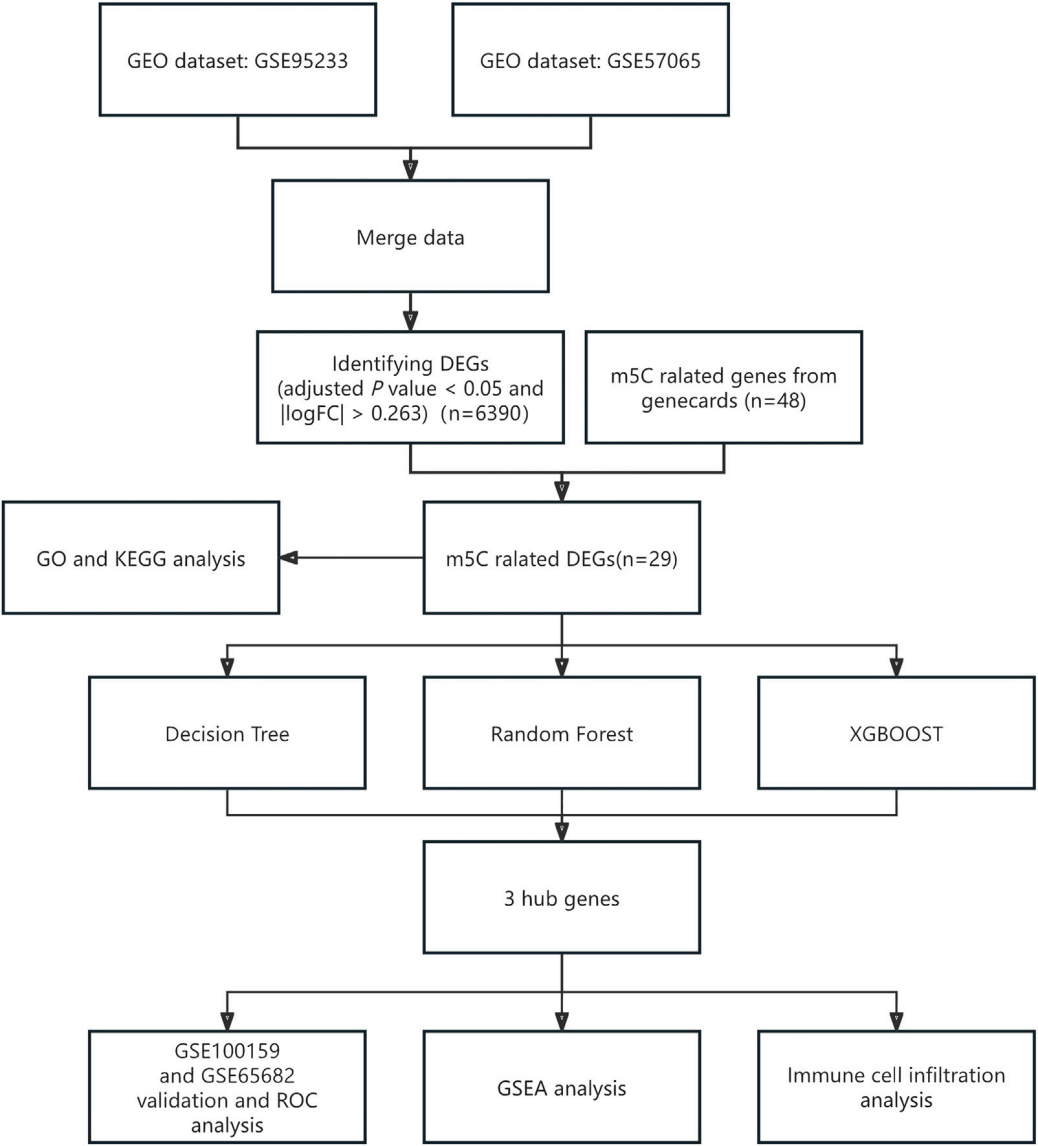
Flowchart of full text analysis. GEO, gene expression omnibus; m5C, 5-methylcytosine; DEGs, differentially expressed genes; GO, gene ontology; GSEA, gene set enrichment analysis; KEGG, Kyoto Encyclopedia of Genes and Genomes; ROC, receiver operating characteristic.

**FIGURE 2:**
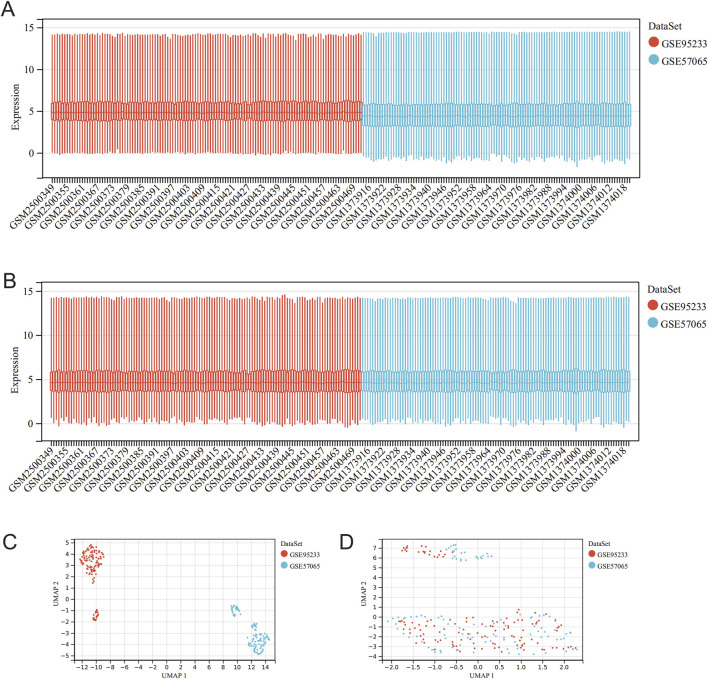
Data set merging and de-batching. **(A)** Box line plot of data distribution comparison before data pooling and removal of batch effect for GSE95233 and GSE57065; **(B)** Box line plot of data distribution comparison after data pooling and removal of batch effect for GSE95233 and GSE57065; **(C)** Umap plot of data distribution comparison before data pooling and removal of batch effect for GSE95233 and GSE57065; **(D)** Umap plot of data distribution comparison between GSE95233 and GSE57065 data set and after removal of batch effect.

Figure 1 shows the full text analysis flow. Figure 2 displays the box-and-line and UMAP plots comparing data distribution before and after batch effect removal for the merged dataset (GSE95233 and GSE57065 merged). The box plots (Figures 2A, B) reveal significant differences in the sample distribution of each dataset prior to batch effect removal, indicating the presence of a batch effect. However, after batch effect removal, the data distribution among datasets becomes more consistent, with the median aligning on a straight line. The UMAP plot (Figures 2C, D) indicates that the samples within each dataset are closely grouped prior to batch effect removal, implying the presence of a batch effect. Subsequently, the samples from each dataset are intermingled post-batch effect removal, indicating successful elimination of the batch effect.

### 3.2 Screening and enrichment analyses of m5C-related DEGs

**FIGURE 3:**
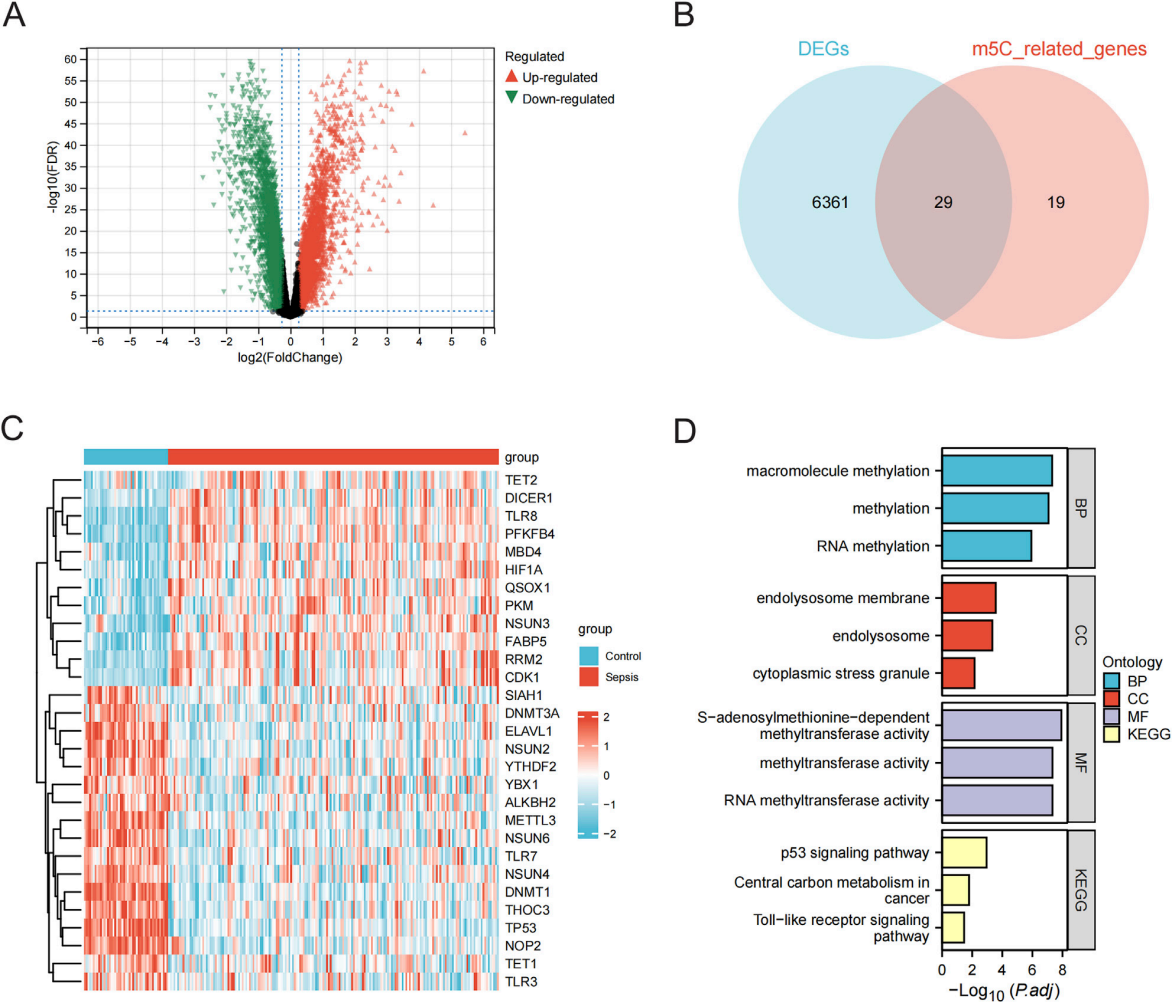
Screening and enrichment analysis of m5C-related DEGs. **(A)** Volcano plot of expression differences between normal and sepsis sample groups in the combined data (GSE95233 and GSE57065 combined) cohort. **(B)** Wayne plots of differential and m5C-related genes. **(C)** Heatmap of m5C-related differential gene expression in the combined data cohort. **(D)** Bar graph of GO and KEGG analysis of m5C-related differential genes. BP, biological processes; CC, cellular components; MF, molecular functions; m5C, 5-methylcytosine; DEGs, differentially expressed genes; KEGG, Kyoto Encyclopedia of Genes and Genomes.

Differential expression analyses of 47 normal samples and 184 sepsis samples from the merged data (GSE95233 and GSE57065 merged) cohort were performed using the limma package, and a total of 6,390 DEGs were identified and plotted in a volcano diagram (Figure 3A). A total of 48 genes associated with m5C were gathered from GeneCards (Supplementary Table 1), and 29 differential genes related to m5C were identified by overlapping the differential genes with m5C-related genes using a Venn diagram (Figure 3B; Supplementary Table 2). Heatmaps for the m5C-related differential genes were then created (Figure 3C). Functional annotation analysis and pathway enrichment analysis were conducted to identify the biological functions of the m5C-related genes using the clusterProfiler package of R. The m5C-related genes were primarily associated with large molecule modification, RNA modification, and cellular stress response. They were also found to be enriched in pathways such as the p53 signaling pathway, central carbon metabolism in cancer, and Toll-like receptor signaling pathway (Figure 3D; Supplementary Table 3).

### 3.3 Three machine learning screens for hub genes

**FIGURE 4:**
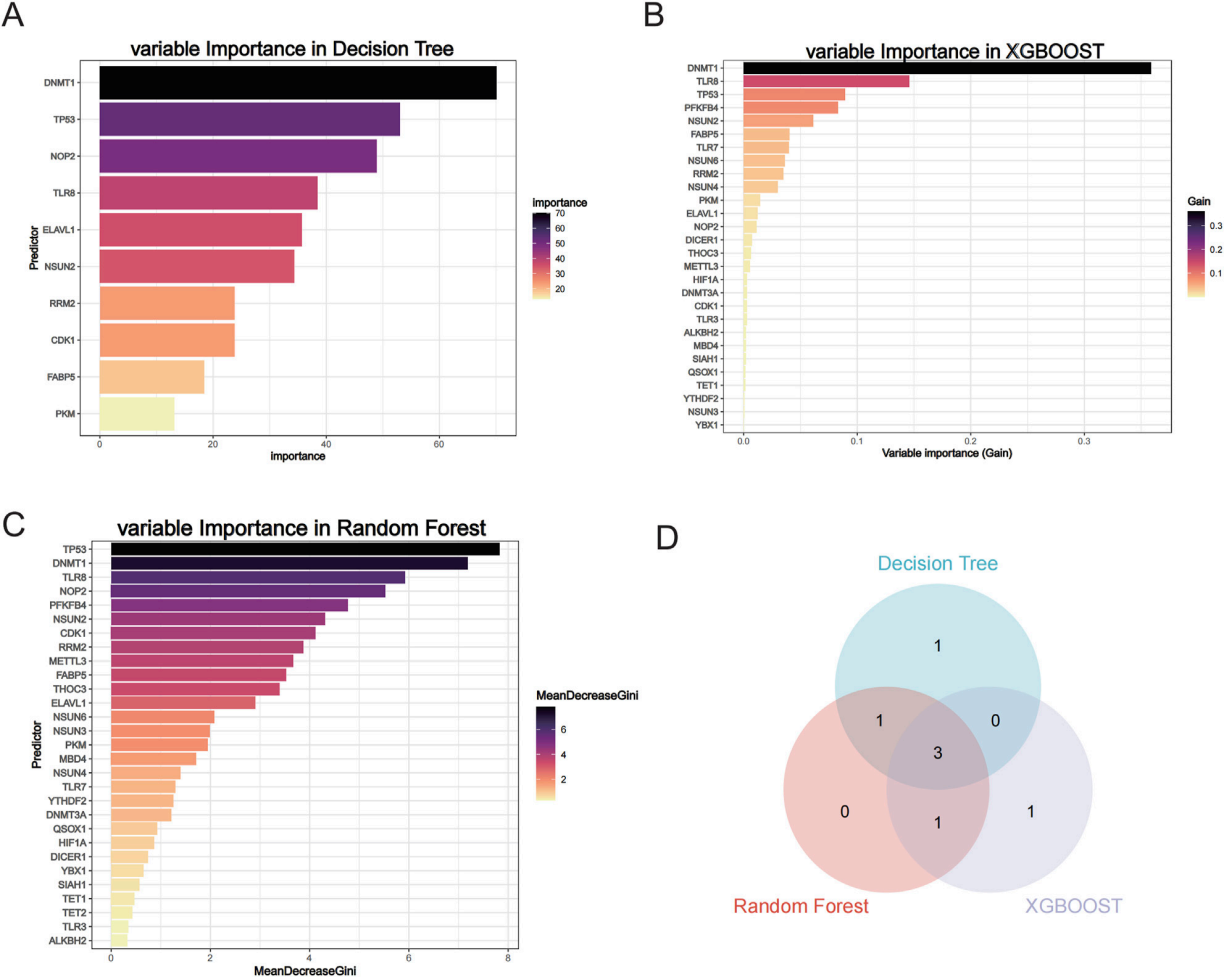
Three machine learning screening hub genes. **(A)** Variable Importance in Decision Tree. **(B)** Variable Importance in XGB00ST. **(C)** Variable Importance in Random Forest. **(D)** Wayne diagram of three machine learning screening hub genes.

We performed three machine learning algorithms to analyze the 29 m5C-related differential genes, including Decision Tree, XGB00ST, and Random Forest, and also showed the variable importance of the different algorithms (Figures 4A–C). To screen the most diagnostically significant genes, we selected only the top 5 genes in importance from each method and used the common genes among them as hub genes, and the genes intersected by the 3 methods were *DNMT1*, *TP53*, and *TLR8* (Figure 4D; Supplementary Table 4).

### 3.4 Validation of hub genes expression differences and diagnostic performance

**FIGURE 5:**
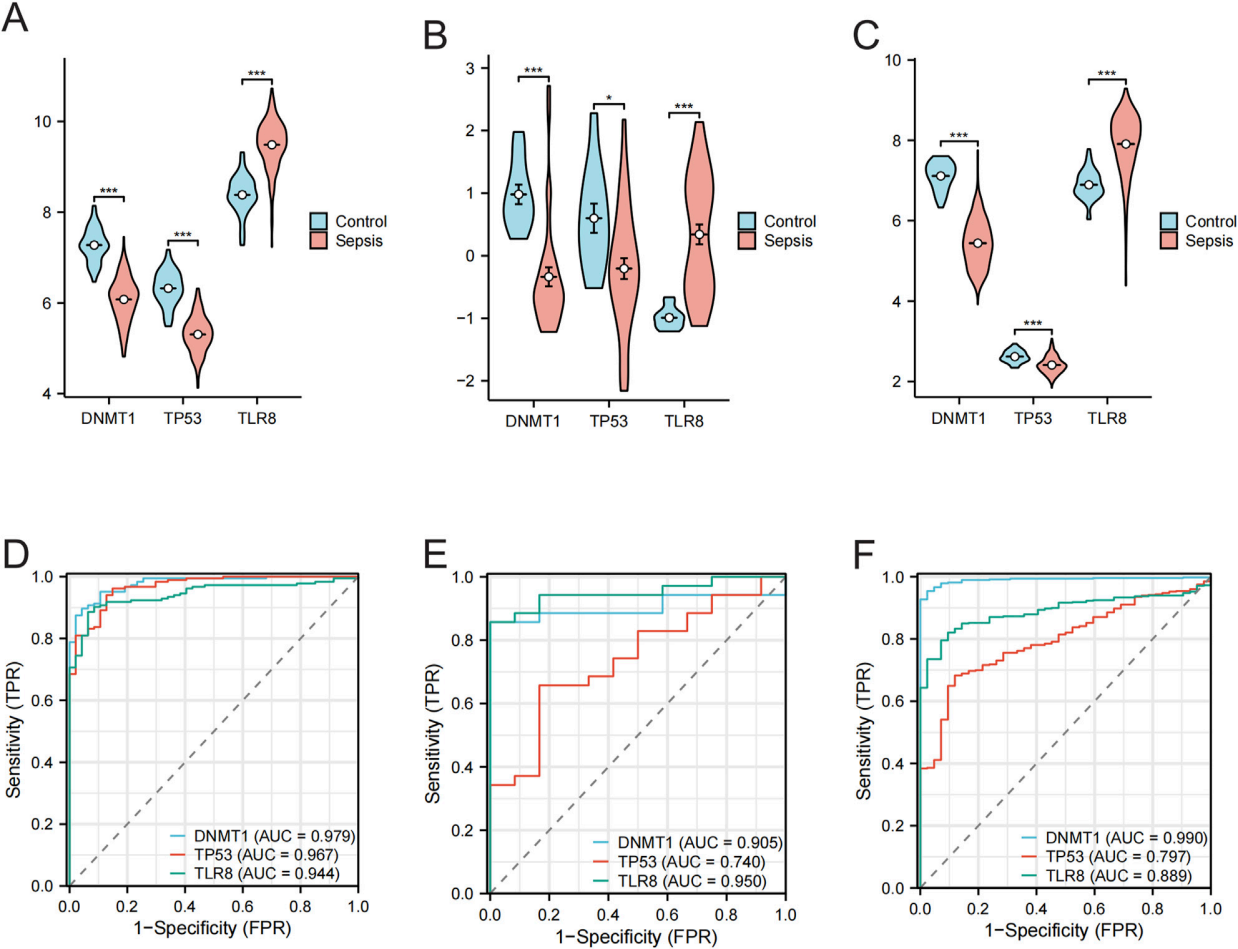
Differential expression of the hub gene in normal and sepsis samples and its ROC analysis results. **(A)** Violin plot showing the expression difference of *DNMT1*, *TP53*, and *TLR8* genes in normal vs sepsis groups in the merged data (GSE95233 and GSE57065 merged). **(B)** Violin plot showing the expression differences of *DNMT1*, *TP53*, and *TLR8* genes in the GSE100159 dataset in the normal group vs the sepsis group. **(C)** Violin plot showing the expression differences of *DNMT1*, *TP53*, and *TLR8* genes in the GSE65682 dataset in the normal group *versus* the sepsis group. **(D)** ROC curves analyzing the diagnostic efficacy of the three hub genes *DNMT1*, *TP53*, and *TLR8* in the combined data (GSE95233 and GSE57065 combined). **(E)** ROC curves analyzing the diagnostic efficacy of the three hub genes *DNMT1*, *TP53*, and *TLR8* in the GSE100159 dataset. **(F)** ROC curves analyzing the diagnostic efficacy of the three hub genes *DNMT1*, *TP53*, and *TLR8* in the GSE65682 dataset.

The merged data from GSE95233 and GSE57065 showed a significant decrease in the expression of *DNMT1* and *TP53* in the sepsis group compared to the control group, while the expression of *TLR8* was significantly elevated in the sepsis group (Figure 5A), and the ROC analysis found the AUC to be 0.979, 0.967, and 0.944 for *DNMT1*, *TP53*, and *TLR8*, respectively (Figure 5D). In the validation set GSE100159, the sepsis group showed significantly decreased levels of *DNMT1* and *TP53* compared to the control group, along with significantly increased levels of *TLR8* (Figure 5B). The AUC values from ROC analysis were 0.905, 0.740, and 0.950 for *DNMT1*, *TP53*, and *TLR8*, respectively (Figure 5E). Within the GSE65682 validation set, the levels of *DNMT1* and *TP53* were notably reduced in the sepsis group compared to the control group, while *TLR8* levels were significantly elevated in the sepsis group (Figure 5C). The ROC analysis indicated AUC values of 0.990, 0.797, and 0.889 for *DNMT1*, *TP53*, and *TLR8*, respectively (Figure 5F). To sum up, in our research, in both the test set and the validation set, *DNMT1*, *TP53*, and *TLR8* exhibited notable variations in expression levels between normal and sepsis sample groups. The AUCs of these three central genes exceeded 0.7, indicating a distinct diagnostic significance.

### 3.5 Single gene enrichment analysis of hub genes

**FIGURE 6:**
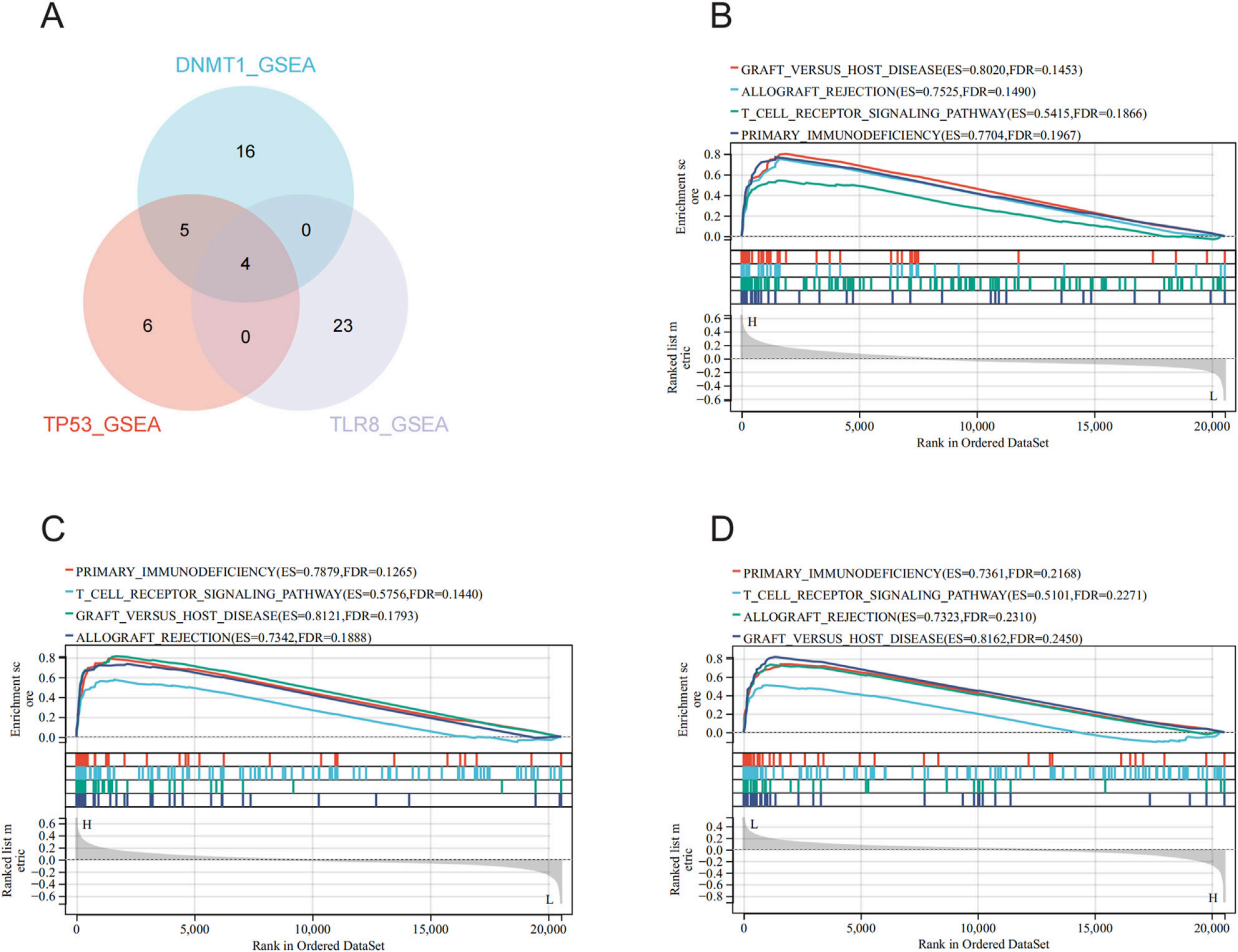
Single gene enrichment analysis and intersection pathway analysis of hub genes. **(A)** Screening of *DNMT1*, *TP53*, and *TLR8* for single gene enrichment analysis of significantly enriched pathways in Wayne plots. **(B)** Single gene enrichment analysis of *DNMT1*. **(C)** Single gene enrichment analysis of *TP53*. **(D)** Single gene enrichment analysis of *TLR8*.

In the merged data (GSE95233 and GSE57065 merged), single-gene GSEA analyses were performed on the three hub genes to obtain the associated pathways for each gene (Figures 6B–D), with 25 significantly enriched pathways by single-gene GSEA analysis for *DNMT1* (Supplementary Table 5), 15 significantly enriched pathways by single-gene GSEA analysis for *TP53* (Supplementary Table 6), and 27 pathways were significantly enriched by single-gene GSEA analysis for *TLR8* (Supplementary Table 7). By intersecting the three hub genes using a Venn diagram (Figure 6A), four common significantly enriched pathways were identified: allograft rejection, graft *versus* host disease, primary immunodeficiency, and t cell receptor signaling pathway. These pathways, crucial for the immune system, indicate that the three hub genes may be pivotal in sepsis development through immune response regulation.

### 3.6 Immune infiltration analysis

**FIGURE 7:**
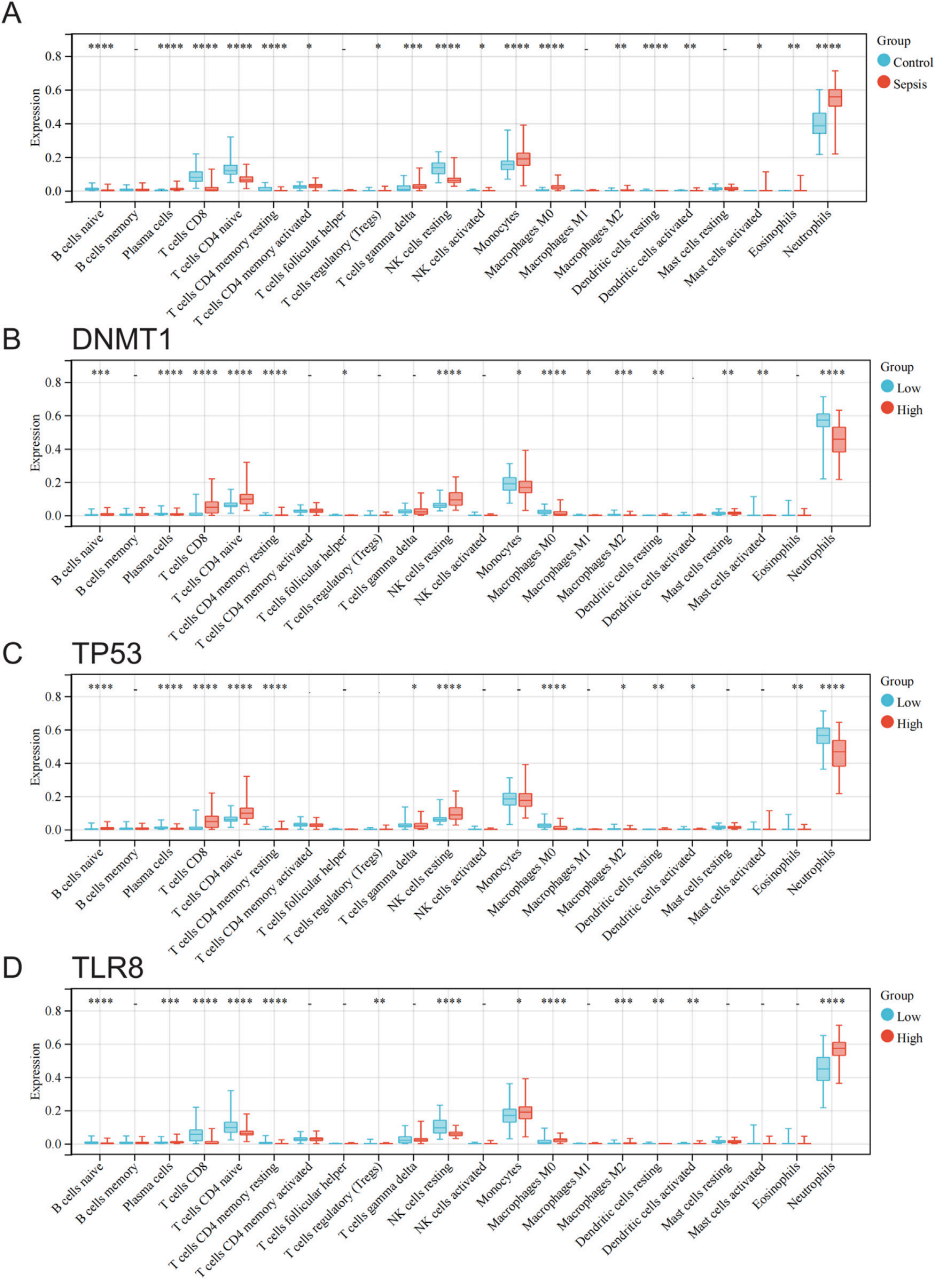
Analysis of differences in immune cell infiltration between groups of normal and sepsis samples and at different hub gene expression levels. **(A)** Differences in immune cell infiltration between normal and sepsis samples. **(B)** Differences in immune cell infiltration between groups with high and low *DNMT1* expression. **(C)** Differences in immune cell infiltration between high and low *TP53* expression groups. **(D)** Differences in immune cell infiltration between high and low *TLR8* expression groups.

The CIBERSORT method was utilized to assess the variations in immune cell penetration between standard samples and sepsis samples. A bar chart was employed to demonstrate the proportion of each kind of immune cell in individual samples (Supplementary Figure 1), while a box plot was created to exhibit the penetration of all immune cells under various grouping conditions (Figure 7). The findings revealed that the immune cells showing distinct penetration levels between standard samples and sepsis samples included B cells naive, plasma cells, T cells CD8, T cells CD4 naive, T cells CD4 memory resting, T cells CD4 memory activated, T cells regulatory (Tregs), T cells gamma delta, NK cells resting, NK cells activated, monocytes, macrophages M0, macrophages M2, dendritic cells resting, dendritic cells activated, mast cells activated, eosinophils, and neutrophils (Figure 7A). The immune cells displaying different penetration levels between the high and low expression groups of *DNMT1* comprised B cells naive, plasma cells, T cells CD8, T cells CD4 naive, T cells CD4 memory resting, T cells follicular helper, NK cells resting, monocytes, macrophages M0, macrophages M1, macrophages M2, dendritic cells resting, mast cells resting, mast cells activated, and neutrophils (Figure 7B). The immune cells manifesting diverse penetration levels between the high and low expression groups of *TP53* encompassed B cells naive, plasma cells, T cells CD8, T cells CD4 naive, T cells CD4 memory resting, T cells gamma delta, NK cells resting, macrophages M0, macrophages M2, dendritic cells resting, dendritic cells activated, eosinophils, and neutrophils (Figure 7C). The immune cells with varying penetration levels between the high and low expression groups of *TLR8* included B cells naive, plasma cells, T cells CD8, T cells CD4 naive, T cells CD4 memory resting, T cells regulatory (Tregs), NK cells resting, monocytes, macrophages M0, macrophages M2, dendritic cells resting, dendritic cells activated, and neutrophils (Figure 7D).

**FIGURE 8:**
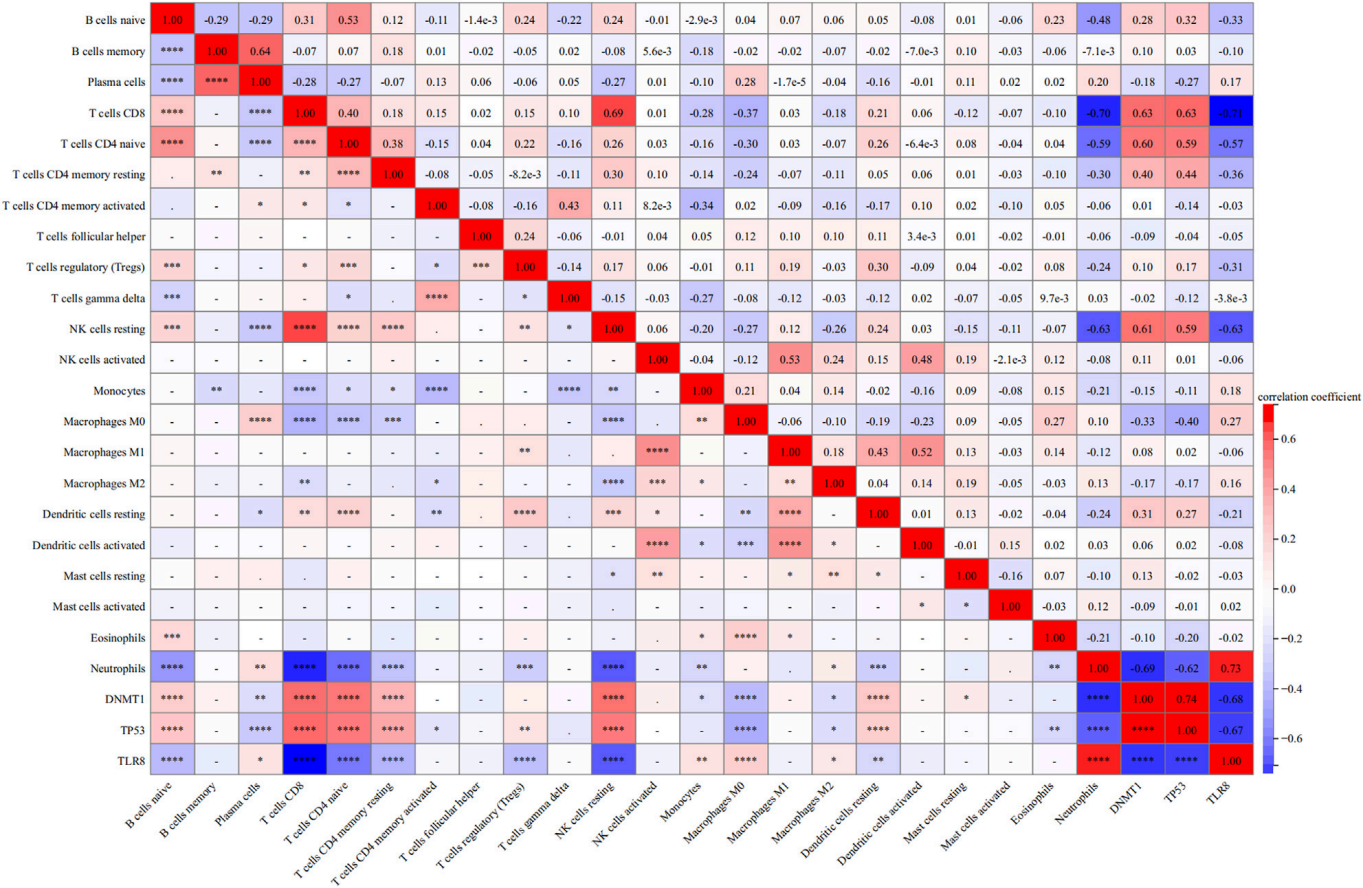
Heatmap of correlation analysis between each immune cell and each hub gene.

**FIGURE 9:**
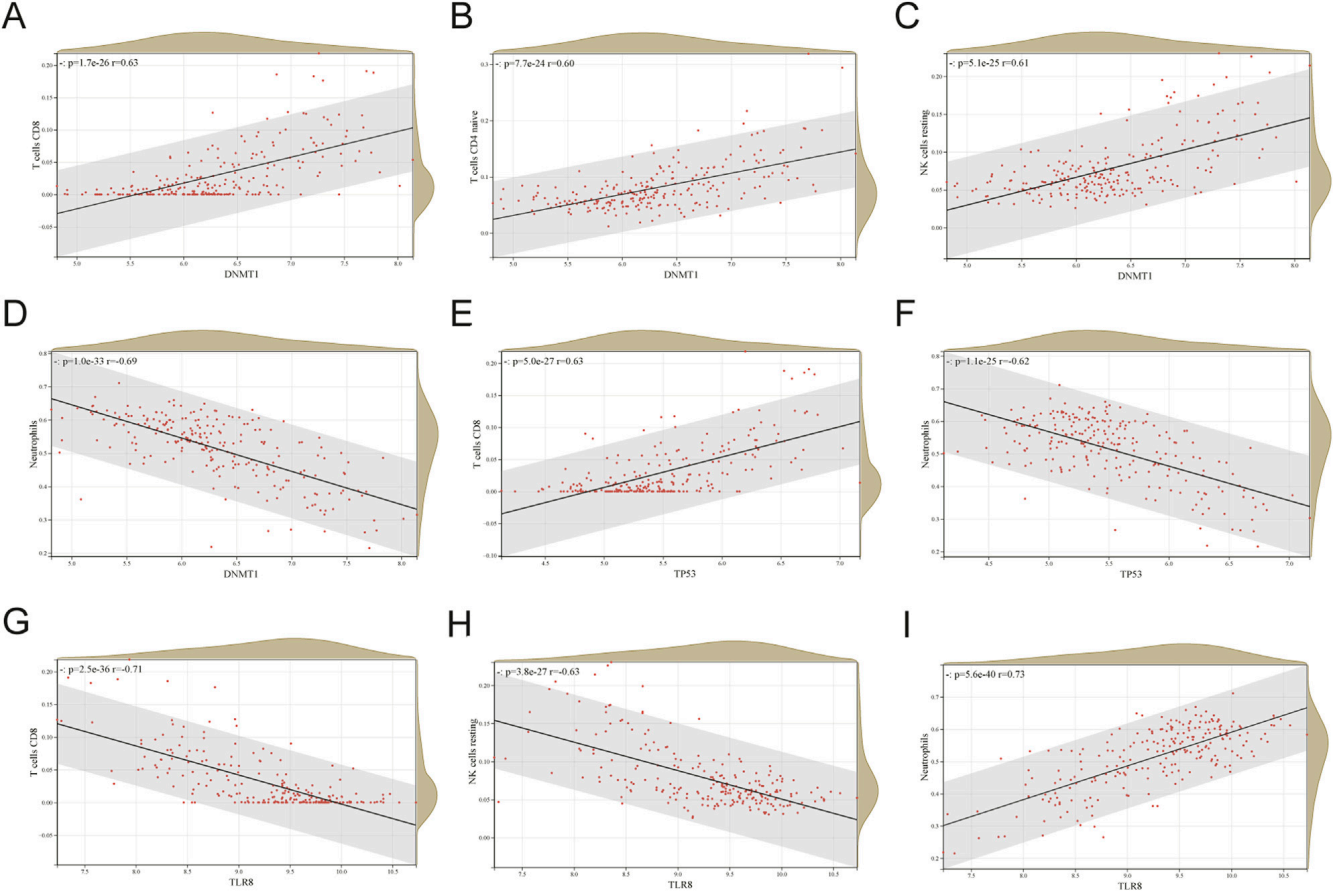
Correlation between each hub gene and specific immune cells. **(A)** *DNMT1* correlation plot with T cells CD8. **(B)** *DNMT1* correlation plot with T cells CD4 naive. **(C)** Correlation plot of *DNMT1* with NK cells resting. **(D)** *DNMT1* correlation plot with neutrophils. **(E)** *TP53* correlation plot with T cells CD8. **(F)** Correlation graph of *TP53* with neutrophils. **(G)** Correlation of *TLR8* with T cells CD8. **(H)** *TLR8* vs NK cells resting correlation graph. **(I)** *TLR8* correlation plot with neutrophils.

Figure 8 displays correlation plots showing the connections between individual hub genes and various types of immune cells. *DNMT1* exhibited strong associations with T cells CD8 (R = 0.63) (Figure 9A), T cells CD4 naive (R = 0.60) (Figure 9B), and NK cells resting (R = 0.61) (Figure 9C), while showing an inverse relationship with neutrophils (R = −0.69) (Figure 9D). *TP53* showed a strong positive association with CD8 T cells (R = 0.63) (Figure 9E) and a negative correlation with neutrophils (R = −0.62) (Figure 9F). *TLR8* exhibited inverse relationships with CD8 T cells (R = −0.71) (Figure 9G) and resting NK cells (R = −0.63) (Figure 9H), while demonstrating a direct correlation with neutrophils (R = 0.73) (Figure 9I).

## 4 Discussion

Sepsis is a serious medical condition caused by an uncontrolled reaction of the body to an infection, resulting in organ failure and a high risk of death (Singer et al., 2016). It poses a significant burden on healthcare systems worldwide, with millions of cases reported annually and substantial healthcare costs (Rudd et al., 2020). The intricate nature of sepsis pathophysiology, which includes complex interactions among the immune system and different organ systems, makes diagnosing and treating it challenging (van der Poll et al., 2017; Cecconi et al., 2018). Early and accurate diagnosis is crucial for improving patient outcomes, yet current diagnostic methods are often insufficiently sensitive or specific (Gotts and Matthay, 2016; Seymour et al., 2016). Hence, it is crucial to develop new indicators and diagnostic instruments to improve the early identification and treatment of sepsis.

The research is centered on exploring the diagnostic capabilities of m5C-associated genes in sepsis and their impact on immune infiltration, with the goal of connecting molecular mechanisms to practical clinical use. Through the integration of various sepsis-related datasets and the utilization of advanced bioinformatics and machine learning methods, we discovered crucial m5C-associated genes that are differentially expressed and identified hub genes that have a strong diagnostic performance (AUC >0.7). These findings were further validated in independent datasets, underscoring their robustness and potential clinical utility. Additionally, the study’s immune infiltration analysis revealed significant correlations between hub genes and immune cell types, providing new insights into the immune regulatory mechanisms in sepsis. This thorough method not only improves our comprehension of sepsis development but also opens up opportunities for creating more accurate diagnostic and treatment plans.

The enrichment analysis results from our study revealed that the m5C-related DEGs are significantly involved in several key biological processes and pathways, including macromolecule methylation, RNA methylation, and the p53 signaling pathway. The results align with earlier research emphasizing the significance of RNA alterations and the p53 pathway in controlling immunity and responding to cellular stress (Vousden and Prives, 2009; Smith and Meissner, 2013; Levine, 2020). Specifically, the p53 signaling pathway is well-known for its role in regulating cell cycle, apoptosis, and genomic stability, which are critical processes in the pathophysiology of sepsis (Vousden and Lane, 2007; Kastenhuber and Lowe, 2017).

*DNMT1*, *TP53*, and *TLR8* were identified as hub genes through our machine learning analysis, and their diagnostic effectiveness was confirmed with AUC values exceeding 0.7. *DNMT1* plays a crucial role in DNA methylation, which is linked to controlling gene activity and immune system reactions (Cedar and Bergman, 2012; Moore et al., 2013). The *TP53* gene, known for suppressing tumors, is essential for responding to cellular stress and has been found to engage with different immune pathways (Vousden and Lane, 2007; Vousden and Prives, 2009). *TLR8* belongs to the Toll-like receptor group, playing a crucial role in identifying pathogens and triggering the body’s natural defense system (Kawai and Akira, 2010; Eigenbrod et al., 2015).

The analysis of a single gene using GSEA showed significant enrichment of *DNMT1*, *TP53*, and *TLR8* in immune-related pathways like allograft rejection. This route plays a role in the body’s defense against transplanted tissues and has similarities with the immune system dysfunction seen in sepsis. The presence of these hub genes in pathways related to the immune system highlights their possible involvement in regulating immune reactions in cases of sepsis.

The analysis of immune cell infiltration showed notable variations in the quantities of different immune cells in normal and sepsis samples, as well as in groups with high and low expression of the hub genes *DNMT1*, *TP53*, and *TLR8*. *DNMT1* and *TP53* showed a strong positive relationship with T cells CD8 (R = 0.63 for both) and a negative relationship with neutrophils (R = −0.69 and R = −0.62, respectively). In contrast, there was an inverse relationship between *TLR8* and CD8 T cells (R = −0.71), while a direct correlation was observed with neutrophils (R = 0.73). These findings are consistent with previous studies that have highlighted the critical role of T cells CD8 and neutrophils in the immune response during sepsis (Hotchkiss and Karl, 2003; Boomer et al., 2011).

T cells CD8 are known for their cytotoxic functions, which are essential for eliminating infected cells and controlling infections (Harty et al., 2000). The connection between *DNMT1* and *TP53* implies that these genes could help boost the cytotoxic reaction, possibly aiding in the elimination of pathogens during sepsis. On the other hand, neutrophils are the first responders to infection and are crucial for the initial immune response (Kolaczkowska and Kubes, 2013; Fine et al., 2020; Richardson et al., 2021). However, their excessive activation can lead to tissue damage and exacerbate sepsis (Kolaczkowska and Kubes, 2013). The negative correlation of *DNMT1* and *TP53* with neutrophils implies a potential regulatory role in mitigating the detrimental effects of neutrophil overactivation (Formosa et al., 2022).

*TLR8*, part of the Toll-like receptor group, plays a role in identifying pathogens and triggering the body’s natural defense system (Kawai and Akira, 2010; Eigenbrod et al., 2015). Its positive correlation with neutrophils and negative correlation with T cells CD8 suggests a complex regulatory mechanism where *TLR8* may enhance the innate immune response while potentially suppressing the adaptive immune response. This dual role could be critical in the context of sepsis, where a balanced immune response is necessary to control infection without causing excessive inflammation (Venet and Monneret, 2018).

Discovering these connections offers fresh perspectives on the immune control processes in sepsis, emphasizing the possibility of *DNMT1*, *TP53*, and *TLR8* as markers for diagnosis and targets for treatment. Comprehending how these hub genes interact with the infiltration of immune cells can assist in creating better approaches for treating sepsis, leading to enhanced results for patients.

Although the results are promising, there are various constraints in this research. Firstly, the research relies solely on bioinformatics analysis without incorporating wet lab experiments, which could provide more direct evidence to support the computational predictions. Secondly, while we utilized large datasets from the GEO database, the absence of specific bioinformatic data on sepsis subtypes prevented a more granular analysis. As a result, we treated sepsis as a homogeneous entity, which is a limitation given the diverse etiologies and mechanisms involved in sepsis. Thirdly, the sample size, although pooled from various datasets, may not fully capture the heterogeneity of sepsis, potentially limiting the generalizability of our findings. Moreover, the lack of clinical validation means that the diagnostic potential of the identified hub genes remains to be confirmed in real-world settings. Finally, while the integration of multiple datasets increased the overall sample size, the possibility of batch effects, despite mitigation efforts using the COMBAT method, may still influence the results and their interpretation.

## 5 Conclusion

In summary, this study successfully identified m5C-related differentially expressed genes in sepsis and highlighted their potential biological functions through GO and KEGG enrichment analyses. Through the utilization of machine learning methods, we pinpointed crucial central genes that exhibit strong diagnostic effectiveness, as illustrated by the analysis of ROC curves and subsequently confirmed in separate datasets. Analysis of gene enrichment and immune infiltration in a single gene shed light on the molecular mechanisms and immune regulatory functions of these central genes in cases of sepsis. Despite the limitations, these findings offer a new perspective on the diagnosis and immune regulation of sepsis, paving the way for future research and potential clinical applications. Future studies should aim to validate these findings through wet-lab experiments and clinical trials to confirm their diagnostic and therapeutic potential.

## Data Availability

The original contributions presented in the study are included in the article/supplementary material, further inquiries can be directed to the corresponding authors.

## Supplementary material

Supplementary Table 1 GeneCards m5C-related genes.

Supplementary Table 2 Expression of 29 m5C-associated differential genes.

Supplementary Table 3 Summary of the top 5 significant entries in the GO and KEGG enrichment analysis results for 29 m5C-related differential genes.

Supplementary Table 4 Introducing 3 hub genes.

Supplementary Table 5 Summary of Significant Enrichment Pathways for Single Gene Enrichment Analysis of DNMT1.

Supplementary Table 6 Summary of Significant Enrichment Pathways for Single Gene Enrichment Analysis of TP53.

Supplementary Table 7 Summary of Significant Enrichment Pathways for Single Gene Enrichment Analysis of TLR8.

Supplementary Figure 1 Histogram of the percentage of each immune cell type in individual samples from the combined data (GSE95233 & GSE57065 combined).
