# Supplementary material for: Identification of m5C-Related gene diagnostic biomarkers for sepsis: a machine learning study
Source: Front Genet. 2024 Oct 30;15:1444003. doi: 10.3389/fgene.2024.1444003 (PMC11558340; doi:10.3389/fgene.2024.1444003)
Supplement: Supplementary file 1 [file Table4.doc]

**Supplementary Table 4 Introducing 3 hub genes.**

| Tag | Full name | Description |
| --- | --- | --- |
| DNMT1 | DNA Methyltransferase 1 | This gene encodes an enzyme that transfers methyl groups to cytosine nucleotides of genomic DNA. This protein is the major enzyme responsible for maintaining methylation patterns following DNA replication and shows a preference for hemi-methylated DNA. |
| TP53 | Tumor Protein P53 | This gene encodes a tumor suppressor protein containing transcriptional activation, DNA binding, and oligomerization domains. The encoded protein responds to diverse cellular stresses to regulate expression of target genes, thereby inducing cell cycle arrest, apoptosis, senescence, DNA repair, or changes in metabolism. |
| TLR8 | Toll Like Receptor 8 | The protein encoded by this gene is a member of the Toll-like receptor (TLR) family which plays a fundamental role in pathogen recognition and activation of innate immunity. They recognize pathogen-associated molecular patterns (PAMPs) that are expressed on infectious agents, and mediate the production of cytokines necessary for the development of effective immunity. |
