# Supplementary material for: Identification of m5C-Related gene diagnostic biomarkers for sepsis: a machine learning study
Source: Front Genet. 2024 Oct 30;15:1444003. doi: 10.3389/fgene.2024.1444003 (PMC11558340; doi:10.3389/fgene.2024.1444003)
Supplement: Supplementary file 2 [file Table6.doc]

**Supplementary Table 6 Summary of Significant Enrichment Pathways for Single Gene Enrichment Analysis of TP53.**

| **Term** | **ES** | **pvalue** | **FDR** |
| --- | --- | --- | --- |
| CELL_ADHESION_MOLECULES_CAMS | 0.545 | 0 | 0.1091 |
| PRIMARY_IMMUNODEFICIENCY | 0.7879 | 0.0019 | 0.1265 |
| NATURAL_KILLER_CELL_MEDIATED_CYTOTOXICITY | 0.5043 | 0.0121 | 0.1282 |
| T_CELL_RECEPTOR_SIGNALING_PATHWAY | 0.5756 | 0 | 0.144 |
| THYROID_CANCER | 0.5018 | 0.0115 | 0.1547 |
| TYPE_I_DIABETES_MELLITUS | 0.7148 | 0.008 | 0.1693 |
| AUTOIMMUNE_THYROID_DISEASE | 0.6586 | 0.0079 | 0.1714 |
| VIRAL_MYOCARDITIS | 0.6423 | 0 | 0.172 |
| GRAFT_VERSUS_HOST_DISEASE | 0.8121 | 0.002 | 0.1793 |
| WNT_SIGNALING_PATHWAY | 0.3356 | 0.0093 | 0.1859 |
| AMINOACYL_TRNA_BIOSYNTHESIS | 0.6815 | 0.0238 | 0.1866 |
| INTESTINAL_IMMUNE_NETWORK_FOR_IGA_PRODUCTION | 0.7277 | 0.0019 | 0.1879 |
| ALLOGRAFT_REJECTION | 0.7342 | 0.0155 | 0.1888 |
| HEMATOPOIETIC_CELL_LINEAGE | 0.55 | 0.0079 | 0.2003 |
| ASTHMA | 0.6921 | 0.0139 | 0.208 |
