# Supplementary material for: Identification of m5C-Related gene diagnostic biomarkers for sepsis: a machine learning study
Source: Front Genet. 2024 Oct 30;15:1444003. doi: 10.3389/fgene.2024.1444003 (PMC11558340; doi:10.3389/fgene.2024.1444003)
Supplement: Supplementary file 3 [file Table7.doc]

**Supplementary Table 7 Summary of Significant Enrichment Pathways for Single Gene Enrichment Analysis of TLR8.**

| **Term** | **ES** | **pvalue** | **FDR** |
| --- | --- | --- | --- |
| ALZHEIMERS_DISEASE | -0.4256 | 0.0396 | 0.175 |
| TYPE_II_DIABETES_MELLITUS | -0.4301 | 0.0223 | 0.1902 |
| INSULIN_SIGNALING_PATHWAY | -0.3484 | 0.0085 | 0.1933 |
| GNRH_SIGNALING_PATHWAY | -0.3376 | 0.0146 | 0.1934 |
| MELANOMA | -0.4059 | 0.0277 | 0.1937 |
| LYSOSOME | -0.3937 | 0.0382 | 0.1939 |
| FC_EPSILON_RI_SIGNALING_PATHWAY | -0.3383 | 0.0433 | 0.1946 |
| GLYCEROPHOSPHOLIPID_METABOLISM | -0.4587 | 0.0019 | 0.196 |
| ACUTE_MYELOID_LEUKEMIA | -0.3968 | 0.044 | 0.1967 |
| TOLL_LIKE_RECEPTOR_SIGNALING_PATHWAY | -0.4207 | 0.044 | 0.1983 |
| RENAL_CELL_CARCINOMA | -0.4128 | 0.0163 | 0.2009 |
| NEUROTROPHIN_SIGNALING_PATHWAY | -0.3277 | 0.0429 | 0.2019 |
| DORSO_VENTRAL_AXIS_FORMATION | -0.4883 | 0.021 | 0.2044 |
| O_GLYCAN_BIOSYNTHESIS | -0.5572 | 0.0019 | 0.2053 |
| ALPHA_LINOLENIC_ACID_METABOLISM | -0.5337 | 0.0496 | 0.208 |
| GLYCOSPHINGOLIPID_BIOSYNTHESIS_LACTO_AND_NEOLACTO_SERIES | -0.549 | 0.0184 | 0.2099 |
| REGULATION_OF_ACTIN_CYTOSKELETON | -0.325 | 0.036 | 0.2108 |
| COMPLEMENT_AND_COAGULATION_CASCADES | -0.5245 | 0.0159 | 0.2115 |
| SPHINGOLIPID_METABOLISM | -0.5564 | 0.0039 | 0.2155 |
| PRIMARY_IMMUNODEFICIENCY | 0.7361 | 0.0175 | 0.2168 |
| STARCH_AND_SUCROSE_METABOLISM | -0.5175 | 0.0182 | 0.2184 |
| PANTOTHENATE_AND_COA_BIOSYNTHESIS | -0.6858 | 0.006 | 0.2225 |
| T_CELL_RECEPTOR_SIGNALING_PATHWAY | 0.5101 | 0.038 | 0.2271 |
| ALLOGRAFT_REJECTION | 0.7323 | 0.0176 | 0.231 |
| ADIPOCYTOKINE_SIGNALING_PATHWAY | -0.3575 | 0.0202 | 0.2328 |
| PPAR_SIGNALING_PATHWAY | -0.4393 | 0.0493 | 0.2345 |
| GRAFT_VERSUS_HOST_DISEASE | 0.8162 | 0.0019 | 0.245 |
