# Supplementary material for: Identification of m5C-Related gene diagnostic biomarkers for sepsis: a machine learning study
Source: Front Genet. 2024 Oct 30;15:1444003. doi: 10.3389/fgene.2024.1444003 (PMC11558340; doi:10.3389/fgene.2024.1444003)
Supplement: Supplementary file 4 [file Table3.doc]

**Supplementary Table 3 Summary of the top 5 significant entries in the GO and KEGG enrichment analysis results for 29 m5C-related differential genes.**

| **Ontology** | **ID** | **Description** | **GeneRatio** | **BgRatio** | **pvalue** | **p.adjust** |
| --- | --- | --- | --- | --- | --- | --- |
| BP | GO:0043414 | macromolecule methylation | 10/29 | 331/18800 | 3.72e-11 | 4.83e-08 |
| BP | GO:0032259 | methylation | 10/29 | 376/18800 | 1.3e-10 | 8.42e-08 |
| BP | GO:0001510 | RNA methylation | 6/29 | 83/18800 | 2.7e-09 | 1.17e-06 |
| BP | GO:0034470 | ncRNA processing | 9/29 | 424/18800 | 9.31e-09 | 3.02e-06 |
| BP | GO:0046700 | heterocycle catabolic process | 9/29 | 443/18800 | 1.36e-08 | 3.31e-06 |
| CC | GO:0036020 | endolysosome membrane | 3/29 | 19/19594 | 2.78e-06 | 0.0003 |
| CC | GO:0036019 | endolysosome | 3/29 | 28/19594 | 9.31e-06 | 0.0005 |
| CC | GO:0010494 | cytoplasmic stress granule | 3/29 | 79/19594 | 0.0002 | 0.0070 |
| CC | GO:0036464 | cytoplasmic ribonucleoprotein granule | 4/29 | 243/19594 | 0.0004 | 0.0105 |
| CC | GO:0035770 | ribonucleoprotein granule | 4/29 | 261/19594 | 0.0006 | 0.0110 |
| MF | GO:0008757 | S-adenosylmethionine-dependent methyltransferase activity | 8/29 | 157/18410 | 8.62e-11 | 1.18e-08 |
| MF | GO:0008173 | RNA methyltransferase activity | 6/29 | 65/18410 | 6.82e-10 | 4.65e-08 |
| MF | GO:0008168 | methyltransferase activity | 8/29 | 214/18410 | 1.02e-09 | 4.65e-08 |
| MF | GO:0016741 | transferase activity, transferring one-carbon groups | 8/29 | 225/18410 | 1.51e-09 | 5.18e-08 |
| MF | GO:0003725 | double-stranded RNA binding | 5/29 | 75/18410 | 1.08e-07 | 2.95e-06 |
| KEGG | hsa04115 | p53 signaling pathway | 4/17 | 73/8164 | 1.28e-05 | 0.0011 |
| KEGG | hsa05230 | Central carbon metabolism in cancer | 3/17 | 70/8164 | 0.0004 | 0.0162 |
| KEGG | hsa04620 | Toll-like receptor signaling pathway | 3/17 | 104/8164 | 0.0012 | 0.0344 |
| KEGG | hsa05206 | MicroRNAs in cancer | 4/17 | 310/8164 | 0.0033 | 0.0705 |
| KEGG | hsa00270 | Cysteine and methionine metabolism | 2/17 | 51/8164 | 0.0049 | 0.0843 |

GO, gene ontology; BP, biological process; CC, cellular component; MF, molecular function; KEGG, kyoto encyclopedia of genes and genomes.
