# Supplementary material for: Identification of m5C-Related gene diagnostic biomarkers for sepsis: a machine learning study
Source: Front Genet. 2024 Oct 30;15:1444003. doi: 10.3389/fgene.2024.1444003 (PMC11558340; doi:10.3389/fgene.2024.1444003)
Supplement: Supplementary file 5 [file Table1.doc]

**Supplementary Table 1 GeneCards m5C-related genes.**

| **Gene Symbol** | **Description** | **Category** | **Relevance score** |
| --- | --- | --- | --- |
| NSUN2 | NOP2/Sun RNA Methyltransferase 2 | Protein Coding | 12.53981781 |
| YBX1 | Y-Box Binding Protein 1 | Protein Coding | 7.700340271 |
| ALYREF | Aly/REF Export Factor | Protein Coding | 5.133355618 |
| NOP2 | NOP2 Nucleolar Protein | Protein Coding | 3.131569386 |
| YTHDF2 | YTH N6-Methyladenosine RNA Binding Protein F2 | Protein Coding | 2.453456163 |
| NSUN3 | NOP2/Sun RNA Methyltransferase 3 | Protein Coding | 2.096771479 |
| ELAVL1 | ELAV Like RNA Binding Protein 1 | Protein Coding | 1.739733219 |
| QSOX1 | Quiescin Sulfhydryl Oxidase 1 | Protein Coding | 1.555469751 |
| TET1 | Tet Methylcytosine Dioxygenase 1 | Protein Coding | 1.522357702 |
| FMR1 | Fragile X Messenger Ribonucleoprotein 1 | Protein Coding | 1.487349033 |
| CDKN1A | Cyclin Dependent Kinase Inhibitor 1A | Protein Coding | 1.366480827 |
| ERCC2 | ERCC Excision Repair 2, TFIIH Core Complex Helicase Subunit | Protein Coding | 1.366480827 |
| METTL3 | Methyltransferase 3, N6-Adenosine-Methyltransferase Complex Catalytic Subunit | Protein Coding | 1.366480827 |
| METTL14 | Methyltransferase 14, N6-Adenosine-Methyltransferase Subunit | Protein Coding | 1.366480827 |
| FABP5 | Fatty Acid Binding Protein 5 | Protein Coding | 1.318312168 |
| NSUN4 | NOP2/Sun RNA Methyltransferase 4 | Protein Coding | 1.318312168 |
| NSUN5 | NOP2/Sun RNA Methyltransferase 5 | Protein Coding | 1.26719439 |
| SUMO2 | Small Ubiquitin Like Modifier 2 | Protein Coding | 1.263638377 |
| SUMO3 | Small Ubiquitin Like Modifier 3 | Protein Coding | 1.263638377 |
| TEAD1 | TEA Domain Transcription Factor 1 | Protein Coding | 0.9102211 |
| NSUN6 | NOP2/Sun RNA Methyltransferase 6 | Protein Coding | 0.706834495 |
| YAP1 | Yes1 Associated Transcriptional Regulator | Protein Coding | 0.499807477 |
| MBD4 | Methyl-CpG Binding Domain 4, DNA Glycosylase | Protein Coding | 0.408091098 |
| AR | Androgen Receptor | Protein Coding | 0.353417248 |
| PKM | Pyruvate Kinase M1/2 | Protein Coding | 0.353417248 |
| DICER1 | Dicer 1, Ribonuclease III | Protein Coding | 0.353417248 |
| HIF1A | Hypoxia Inducible Factor 1 Subunit Alpha | Protein Coding | 0.353417248 |
| TET2 | Tet Methylcytosine Dioxygenase 2 | Protein Coding | 0.353417248 |
| TRDMT1 | TRNA Aspartic Acid Methyltransferase 1 | Protein Coding | 0.353417248 |
| DUS3L | Dihydrouridine Synthase 3 Like | Protein Coding | 0.353417248 |
| TP53 | Tumor Protein P53 | Protein Coding | 0.288563997 |
| FOXC2 | Forkhead Box C2 | Protein Coding | 0.288563997 |
| TDG | Thymine DNA Glycosylase | Protein Coding | 0.288563997 |
| TRMT2A | TRNA Methyltransferase 2 Homolog A | Protein Coding | 0.288563997 |
| ALKBH2 | AlkB Homolog 2, Alpha-Ketoglutarate Dependent Dioxygenase | Protein Coding | 0.288563997 |
| TRMT2B | TRNA Methyltransferase 2 Homolog B | Protein Coding | 0.288563997 |
| DNMT1 | DNA Methyltransferase 1 | Protein Coding | 0.204045549 |
| DNMT3A | DNA Methyltransferase 3 Alpha | Protein Coding | 0.204045549 |
| DNMT3B | DNA Methyltransferase 3 Beta | Protein Coding | 0.204045549 |
| TLR3 | Toll Like Receptor 3 | Protein Coding | 0.204045549 |
| TLR7 | Toll Like Receptor 7 | Protein Coding | 0.204045549 |
| TLR8 | Toll Like Receptor 8 | Protein Coding | 0.204045549 |
| CDK1 | Cyclin Dependent Kinase 1 | Protein Coding | 0.204045549 |
| RRM2 | Ribonucleotide Reductase Regulatory Subunit M2 | Protein Coding | 0.204045549 |
| SIAH1 | Siah E3 Ubiquitin Protein Ligase 1 | Protein Coding | 0.204045549 |
| CSF2 | Colony Stimulating Factor 2 | Protein Coding | 0.204045549 |
| PFKFB4 | 6-Phosphofructo-2-Kinase/Fructose-2,6-Biphosphatase 4 | Protein Coding | 0.204045549 |
| THOC3 | THO Complex Subunit 3 | Protein Coding | 0.204045549 |
