# Supplementary material for: Identification of m5C-Related gene diagnostic biomarkers for sepsis: a machine learning study
Source: Front Genet. 2024 Oct 30;15:1444003. doi: 10.3389/fgene.2024.1444003 (PMC11558340; doi:10.3389/fgene.2024.1444003)
Supplement: Supplementary file 6 [file Table5.doc]

**Supplementary Table 5 Summary of Significant Enrichment Pathways for Single Gene Enrichment Analysis of DNMT1.**

| **Term** | **ES** | **pvalue** | **FDR** |
| --- | --- | --- | --- |
| RNA_POLYMERASE | 0.6307 | 0.0019 | 0.0974 |
| MISMATCH_REPAIR | 0.6925 | 0.0339 | 0.1412 |
| GLYCOSYLPHOSPHATIDYLINOSITOL_GPI_ANCHOR_BIOSYNTHESIS | 0.5973 | 0.0217 | 0.1418 |
| NUCLEOTIDE_EXCISION_REPAIR | 0.575 | 0.036 | 0.1448 |
| TYPE_I_DIABETES_MELLITUS | 0.6912 | 0.0202 | 0.1452 |
| N_GLYCAN_BIOSYNTHESIS | 0.5267 | 0.0495 | 0.1452 |
| GRAFT_VERSUS_HOST_DISEASE | 0.802 | 0.01 | 0.1453 |
| CYSTEINE_AND_METHIONINE_METABOLISM | 0.4865 | 0.0452 | 0.149 |
| ALLOGRAFT_REJECTION | 0.7525 | 0.008 | 0.149 |
| ANTIGEN_PROCESSING_AND_PRESENTATION | 0.6518 | 0.0241 | 0.1503 |
| LIMONENE_AND_PINENE_DEGRADATION | 0.6232 | 0.0343 | 0.1584 |
| VIRAL_MYOCARDITIS | 0.5826 | 0.014 | 0.1597 |
| INTESTINAL_IMMUNE_NETWORK_FOR_IGA_PRODUCTION | 0.6768 | 0.0178 | 0.1604 |
| AMINOACYL_TRNA_BIOSYNTHESIS | 0.756 | 0 | 0.1679 |
| SPLICEOSOME | 0.5406 | 0.0452 | 0.1692 |
| BUTANOATE_METABOLISM | 0.4803 | 0.0258 | 0.171 |
| PYRIMIDINE_METABOLISM | 0.5183 | 0.0291 | 0.1724 |
| BASE_EXCISION_REPAIR | 0.666 | 0.002 | 0.1747 |
| T_CELL_RECEPTOR_SIGNALING_PATHWAY | 0.5415 | 0.0299 | 0.1866 |
| DNA_REPLICATION | 0.7486 | 0.0213 | 0.1924 |
| COLORECTAL_CANCER | 0.4189 | 0.0469 | 0.1925 |
| PURINE_METABOLISM | 0.4355 | 0.008 | 0.1933 |
| ASTHMA | 0.6851 | 0.0323 | 0.1948 |
| PRIMARY_IMMUNODEFICIENCY | 0.7704 | 0.0039 | 0.1967 |
| PYRUVATE_METABOLISM | 0.4303 | 0.0359 | 0.1982 |
