# Supplementary figures and images for: Identification of m5C-Related gene diagnostic biomarkers for sepsis: a machine learning study
Source: Front Genet. 2024 Oct 30;15:1444003. doi: 10.3389/fgene.2024.1444003 (PMC11558340; doi:10.3389/fgene.2024.1444003)

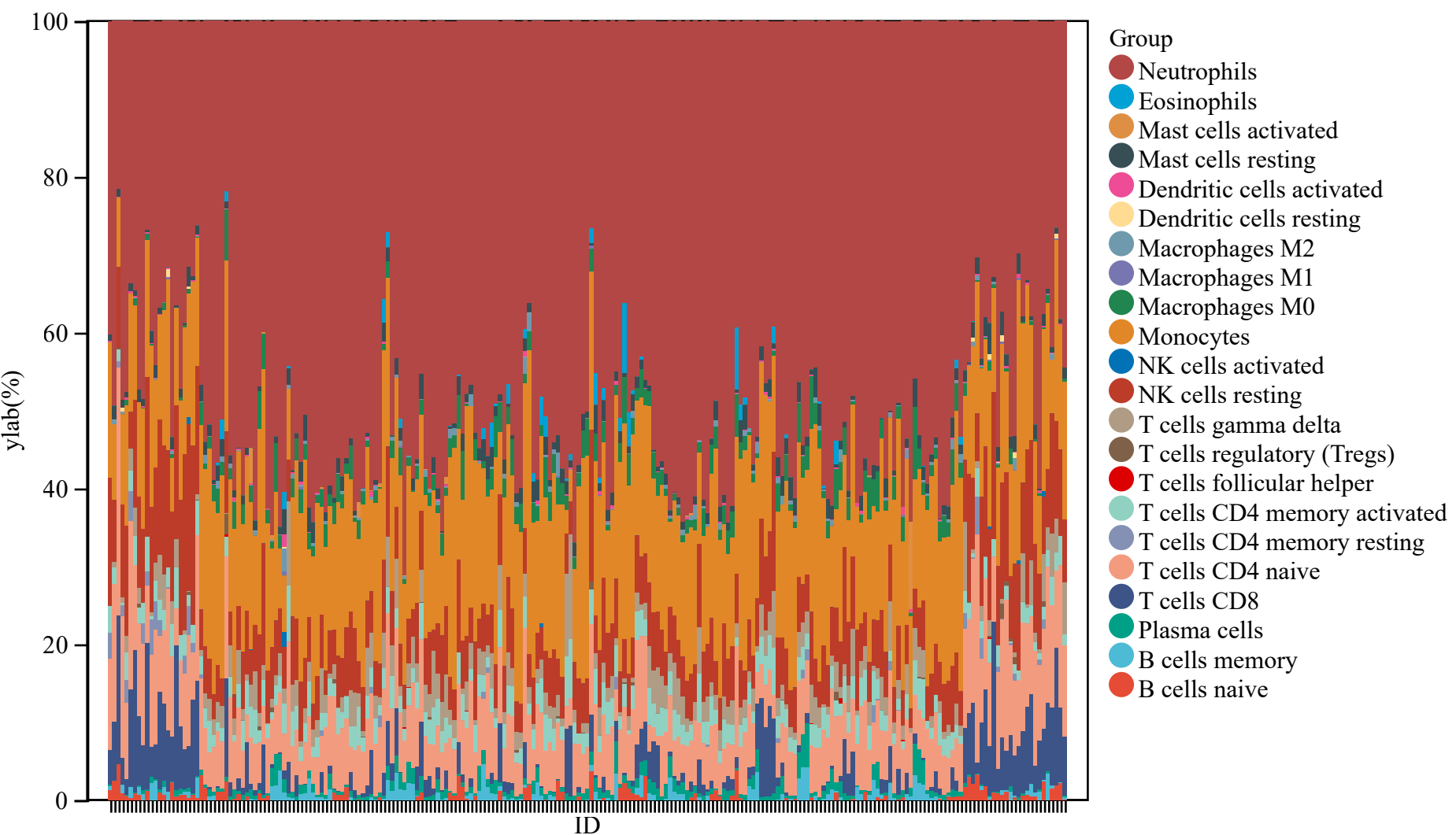

Supplement: Supplementary file 7 [file DataSheet1.pdf]
