# Supplementary material for: Identification of m5C-Related gene diagnostic biomarkers for sepsis: a machine learning study
Source: Front Genet. 2024 Oct 30;15:1444003. doi: 10.3389/fgene.2024.1444003 (PMC11558340; doi:10.3389/fgene.2024.1444003)
Supplement: Supplementary file 8 [file Table2.doc]

**Supplementary Table 2 Expression of 29 m5C-associated differential genes.**

| [**Symbol**](https://www.genecards.org/Search/Keyword?queryString=THOC3&pageSize=25&startPage=0&sort=Symbol&sortDir=Ascending) | **Description** | **logFC** | **adj.P.Val** | **Up/Down** |
| --- | --- | --- | --- | --- |
| TP53 | Tumor Protein P53 | -1.016287173 | 5.71E-37 | down |
| DNMT1 | DNA Methyltransferase 1 | -1.195398903 | 6.03E-37 | down |
| NOP2 | NOP2 nucleolar protein | -0.775328932 | 7.31E-32 | down |
| TLR8 | Toll Like Receptor 8 | 1.103606394 | 1.49E-28 | up |
| PFKFB4 | 6-Phosphofructo-2-Kinase/Fructose-2,6-Biphosphatase 4 | 0.561071406 | 1.15E-25 | up |
| NSUN2 | NOP2/Sun RNA Methyltransferase 2 | -0.584605277 | 2.84E-24 | down |
| ELAVL1 | ELAV Like RNA Binding Protein 1 | -0.46255692 | 2.08E-22 | down |
| METTL3 | Methyltransferase 3, N6-Adenosine-Methyltransferase Complex Catalytic Subunit | -0.475113841 | 4.92E-22 | down |
| THOC3 | THO Complex Subunit 3 | -0.779429904 | 6.01E-22 | down |
| FABP5 | Fatty Acid Binding Protein 5 | 0.940331325 | 3.98E-21 | up |
| NSUN6 | NOP2/Sun RNA Methyltransferase 6 | -0.464940412 | 1.04E-18 | down |
| RRM2 | Ribonucleotide Reductase Regulatory Subunit M2 | 1.79931078 | 3.40E-16 | up |
| MBD4 | Methyl-CpG Binding Domain 4, DNA Glycosylase | 0.494364907 | 9.56E-16 | up |
| NSUN3 | NOP2/Sun RNA Methyltransferase 3 | 0.399593426 | 8.42E-15 | up |
| NSUN4 | NOP2/Sun RNA Methyltransferase 4 | -0.366250513 | 2.09E-14 | down |
| CDK1 | Cyclin Dependent Kinase 1 | 0.964666665 | 3.28E-14 | up |
| QSOX1 | Quiescin Sulfhydryl Oxidase 1 | 0.409275966 | 5.20E-14 | up |
| PKM | Pyruvate Kinase M1/2 | 0.521013089 | 5.50E-13 | up |
| HIF1A | Hypoxia Inducible Factor 1 Subunit Alpha | 0.656447974 | 1.11E-12 | up |
| YTHDF2 | YTH N6-Methyladenosine RNA Binding Protein F2 | -0.322413645 | 2.83E-12 | down |
| TLR7 | Toll Like Receptor 7 | -0.54849402 | 6.86E-12 | down |
| DNMT3A | DNA Methyltransferase 3 Alpha | -0.325356499 | 1.61E-10 | down |
| DICER1 | Dicer 1, Ribonuclease III | 0.352651864 | 1.48E-08 | up |
| YBX1 | Y-Box Binding Protein 1 | -0.470714882 | 5.99E-08 | down |
| ALKBH2 | AlkB Homolog 2, Alpha-Ketoglutarate Dependent Dioxygenase | -0.29874904 | 6.61E-08 | down |
| TET1 | Tet Methylcytosine Dioxygenase 1 | -0.321329605 | 2.57E-07 | down |
| TLR3 | Toll Like Receptor 3 | -0.304077268 | 2.83E-07 | down |
| SIAH1 | Siah E3 Ubiquitin Protein Ligase 1 | -0.265913249 | 9.91E-07 | down |
| TET2 | Tet Methylcytosine Dioxygenase 2 | 0.479765239 | 3.18E-06 | up |
